# Cancer Clinician Experiences With Telehealth Across Oncology Specialties: Cross-Sectional Survey Study

**DOI:** 10.2196/94610

**Published:** 2026-08-19

**Authors:** Benjamin Baker, Sakshith Reddy Chintala, Sarah Azari, James D Murphy, Hala Madanat, Maria Elena Martinez, Richard M Cripps, Aditya Bagrodia, Humberto Parada Jr, Juan Javier-DesLoges

**Affiliations:** 1 Department of Urology, University of California, San Diego, 200 W. Arbor Drive #8897, San Diego, CA, 92103-8897, United States, 1 (619) 543-2626; 2 Department of Radiation Medicine and Applied Sciences, Unviersity of California San Deigo, , La Jolla, CA, United States; 3 Department of Biology, San Diego State University, San Diego, CA, United States; 4 Herbert Wertheim School of Public Health and Moores Cancer Center, University of California San Diego, La Jolla, CA, United States; 5 Department of Epidemiology and Biostatistics, School of Public Health, San Diego State University, San Diego, CA, United States

**Keywords:** telehealth, oncology, telemedicine, digital health, cancer care delivery, clinician experience

## Abstract

**Background:**

Telehealth use expanded rapidly during the COVID-19 pandemic and has since become integrated into routine oncology care. However, clinician experiences and confidence with telehealth may vary across oncology subspecialties, and these differences remain incompletely characterized.

**Objective:**

This study aimed to evaluate telehealth perceptions, decision-making factors, and confidence across oncology clinicians from multiple specialties at a single academic cancer center.

**Methods:**

We conducted a cross-sectional survey of oncology clinicians at the University of California San Diego Moores Cancer Center. A 27-item Qualtrics questionnaire was distributed by email to attending physicians, fellows, and advanced practice providers across surgical, medical, radiation, and palliative oncology. Survey domains included telehealth coordination, technical quality, communication effectiveness, confidence in remote physical examination, and factors influencing visit modality selection. Quantitative responses were summarized descriptively, and open-ended responses underwent thematic analysis.

**Results:**

Among 114 invited clinicians, 57 (50%) complete responses were analyzed. Among 57 respondents, 86% (n=49) were physicians. Specialties included surgical oncology (n=21, 36.8%), medical oncology (n=18, 31.6%), radiation oncology (n=16, 28.1%), and palliative care (n=2, 3.5%). Telehealth coordination was rated easy or very easy by 78.9% (n=45) of clinicians. Audio and video quality were rated good or very good by 71.9% (n=41) and 57.9% (n=33), respectively. Technical issues prompted at least occasional conversion to audio-only visits for 86% (n=49) of respondents, and 17.5% (n=10) of clinicians reported frequent conversion. Communication satisfaction was high (n=48, 84.2%), yet only 50.9% (n=29) reported moderate or high confidence in performing a virtual physical examination. The need for physical examination (n=53, 93%) and patient travel distance (n=45, 78.9%) were the most influential factors in offering telehealth visits. Confidence varied by specialty and clinical phase: moderate or high confidence was reported by surgical oncology clinicians in 52.4% (11/21) of preoperative and 71.4% (15/21) of postoperative responses, by medical oncology clinicians in 77.8% (14/18) of prechemotherapy and 76.5% (13/17) of during chemotherapy responses, and by radiation oncology clinicians in 87.5% (14/16) of preradiation and 93.8% (15/16) of during radiation responses.

**Conclusions:**

Oncology clinicians reported generally positive experiences with telehealth, although confidence and use patterns varied across specialties and phases of care. Telehealth is viewed as a complementary modality when physical examination is not essential. Improvements in technical reliability and interpreter integration may enhance equitable access and support continued telehealth use in oncology care.

## Introduction

Telehealth use expanded rapidly during the COVID-19 pandemic and has remained integrated into many areas of routine clinical care [1-3]. In oncology, telehealth has particular appeal because patients often require longitudinal care, frequent follow-up, multidisciplinary coordination, and review of laboratory, imaging, pathology, and treatment-related information. For selected visits, virtual care may reduce travel burden, decrease time away from work or caregiving responsibilities, and improve access for patients who live far from cancer centers [1,2].

Despite these advantages, telehealth in oncology is not uniformly applicable across all clinical settings. Cancer care often depends on physical examination, assessment of treatment toxicity, symptom evaluation, shared decision-making, and coordination across medical, surgical, radiation, and supportive care teams. The relative importance of these factors may differ substantially by oncology subspecialty and by phase of care. For example, surgical oncologists may have different levels of confidence in telehealth before versus after an operation, whereas medical and radiation oncologists may use telehealth differently during treatment planning, treatment monitoring, and survivorship care. Prior studies have described telehealth implementation in cancer centers, transition to telehealth in surgical practice, and clinician attitudes toward telehealth in multidisciplinary cancer care, but specialty-specific clinician confidence across different phases of oncology care remains incompletely characterized [4-6].

Understanding clinician experience is important because clinicians play a central role in determining whether telehealth is offered, whether it is considered clinically appropriate, and how technical or communication barriers are managed. Clinician confidence may be shaped by visit type, need for physical examination, institutional technology, interpreter availability, patient age, and other patient-specific factors. Broader telehealth use data also suggest that adoption and experience may be shaped by patient characteristics, clinical setting, and health system infrastructure [7]. These issues are especially relevant because telehealth may improve access for some patients while potentially widening disparities for others if language, technology, or workflow barriers are not addressed.

However, limited data describe how oncology clinicians across different specialties perceive telehealth, what factors influence their decision to offer virtual visits, and how confident they are using telehealth during different phases of cancer care. To address this gap, we conducted a cross-sectional survey of medical, surgical, radiation, and palliative oncology clinicians at a single academic cancer center. We evaluated clinician experiences with telehealth, factors influencing visit modality selection, perceived barriers and facilitators, and confidence in telehealth across specialty-specific clinical scenarios.

## Methods

### Study Design and Setting

We conducted a cross-sectional survey study of oncology clinicians at the University of California San Diego Moores Cancer Center, a National Cancer Institute–designated academic cancer center. The survey was designed to assess clinician experiences with telehealth across multiple oncology subspecialties, including perceived visit quality, technical challenges, communication, confidence in remote assessment, and factors influencing visit modality selection.

### Participants and Recruitment

Medical, surgical, palliative, and radiation oncology physicians, fellows, and advanced practice clinicians were invited to participate by email. The survey was distributed using a combination of individual provider email lists and specialty or institutional email distribution lists. A total of 114 providers were invited, including 32 (28.1%) surgical oncology clinicians, 49 (43%) medical oncology clinicians, 26 (22.8%) radiation oncology clinicians, and 7 (6.1%) palliative care clinicians. The survey was open from October 1, 2024, through January 31, 2025. Participation was voluntary, and no incentive was provided. Responses were treated confidentially and reported in aggregate. The primary analytic cohort included respondents with complete survey data for the main telehealth experience items. Blank or incomplete survey records were excluded from the primary analysis.

### Telehealth Context

At the time of the survey, telehealth visits at our institution were primarily conducted through Epic or MyChart video visits. Telephone visits were used when audiovisual connection could not be established or when technical issues required conversion from video to audio-only care. Survey items therefore focused primarily on video-based telehealth, while also capturing the frequency of conversion to audio-only visits due to technical difficulties.

### Survey Instrument Development

The survey instrument was developed by the study investigators and was administered using Qualtrics. The survey was generally inspired by previously published surveys evaluating telehealth experiences, satisfaction, communication, and technology use [8-11]. Items were newly adapted for oncology-specific workflows, including visit modality decision-making, perceived adequacy of remote physical examination, and clinician confidence across phases of cancer care, such as preoperative and postoperative care, chemotherapy, and radiation treatment. The final instrument included 27 closed- and open-ended items and is provided as Multimedia Appendix 1. The survey was reviewed by study investigators with expertise in oncology, telehealth, and cancer care delivery for face validity, clinical relevance, and clarity before distribution. Formal pilot testing, cognitive interviewing, psychometric validation, and reliability testing were not performed.

### Quantitative Analysis

Descriptive statistics were used to summarize respondent characteristics and survey responses. Categorical variables were reported as counts and percentages. Continuous variables were summarized using mean and SD or median and IQR, as appropriate. For key proportions, 95% CIs were calculated using the Wilson method. Likert-scale responses were collapsed for selected analyses to improve interpretability; responses indicating at least moderate confidence were grouped as moderate or high confidence.

Exploratory chi-square tests were used to compare collapsed confidence proportions across specialty groups, McNemar tests were used for paired within-specialty comparisons across phase-of-care items, and a Friedman test was used to evaluate clinician likelihood of offering telehealth across increasing patient age categories. Given the modest sample size and exploratory design, statistical comparisons were interpreted cautiously.

### Qualitative Analysis

Open-ended responses were analyzed using manual inductive coding informed by grounded theory principles. Two investigators independently reviewed and coded the open-ended responses. Codes were compared, and disagreements were resolved through consensus.

Recurring concepts related to telehealth decision-making, barriers, facilitators, and clinician experience were grouped into themes when similar concepts were identified across responses from at least 2 clinicians. Themes were then reviewed and refined to ensure that they reflected the underlying responses and were clinically interpretable. Representative quotations were selected to illustrate major themes. Qualitative analysis was conducted using exported survey responses rather than dedicated qualitative analysis software.

### Ethical Considerations

This study was approved by the University of California San Diego Institutional Review Board (IRB 810808). Consent information was included within the survey, and respondents were required to proceed through the consent portion before accessing the survey. Participation was voluntary, no incentive was provided, and responses were treated confidentially and reported in aggregate.

## Results

### Respondent Characteristics

A total of 114 clinicians were invited to participate, including 32 (28.1%) surgical oncology clinicians, 49 (43%) medical oncology clinicians, 26 (22.8%) radiation oncology clinicians, and 7 (6.1%) palliative care clinicians. Overall, 65 (50%) survey records were captured, of which 57 (87.7%) were complete and included in the primary analysis, yielding a complete response rate of 50% (57/114). Eight blank or incomplete records were excluded.

Among the 57 respondents, 49 (86%) were physicians and 8 (14%) were advanced practice clinicians. Respondents included surgical oncology clinicians (21/57, 36.8%), medical oncology clinicians (18/57, 31.6%), radiation oncology clinicians (16/57, 28.1%), and palliative care clinicians (2/57, 3.5%). Specialty-specific complete response rates were 65.6% (21/32) for surgical oncology, 36.7% (18/49) for medical oncology, 61.5% (16/26) for radiation oncology, and 28.6% (2/7) for palliative care. Mean respondent age was 46.3 (SD 8.7) years. Most respondents were male (34/57, 59.6%) and non-Hispanic (54/57, 94.7%). Full demographic and professional characteristics are shown in Table 1.

**Table 1.** Demographic and professional characteristics of oncology clinicians participating in the telehealth perceptions survey (N=57).

Characteristics	Participants
Age (y), mean (SD)	46.3 (8.7)
Sex, n (%)	
Male	34 (59.6)
Female	23 (40.4)
Race, n (%)	
Asian	12 (21.1)
Black or African American	4 (7)
Native Hawaiian or Pacific Islander	1 (1.8)
White	35 (61.4)
Other	5 (8.8)
Ethnicity, n (%)	
Hispanic	3 (5.3)
Non-Hispanic	54 (94.7)
Professional role, n (%)	
Physician	49 (86)
Advanced practice provider	8 (14)
Specialty, n (%)	
Medical oncology	18 (31.6)
Palliative care	2 (3.5)
Radiation oncology	16 (28.1)
Surgical oncology	21 (36.8)

### Experience With Telehealth

**Figure 1.**
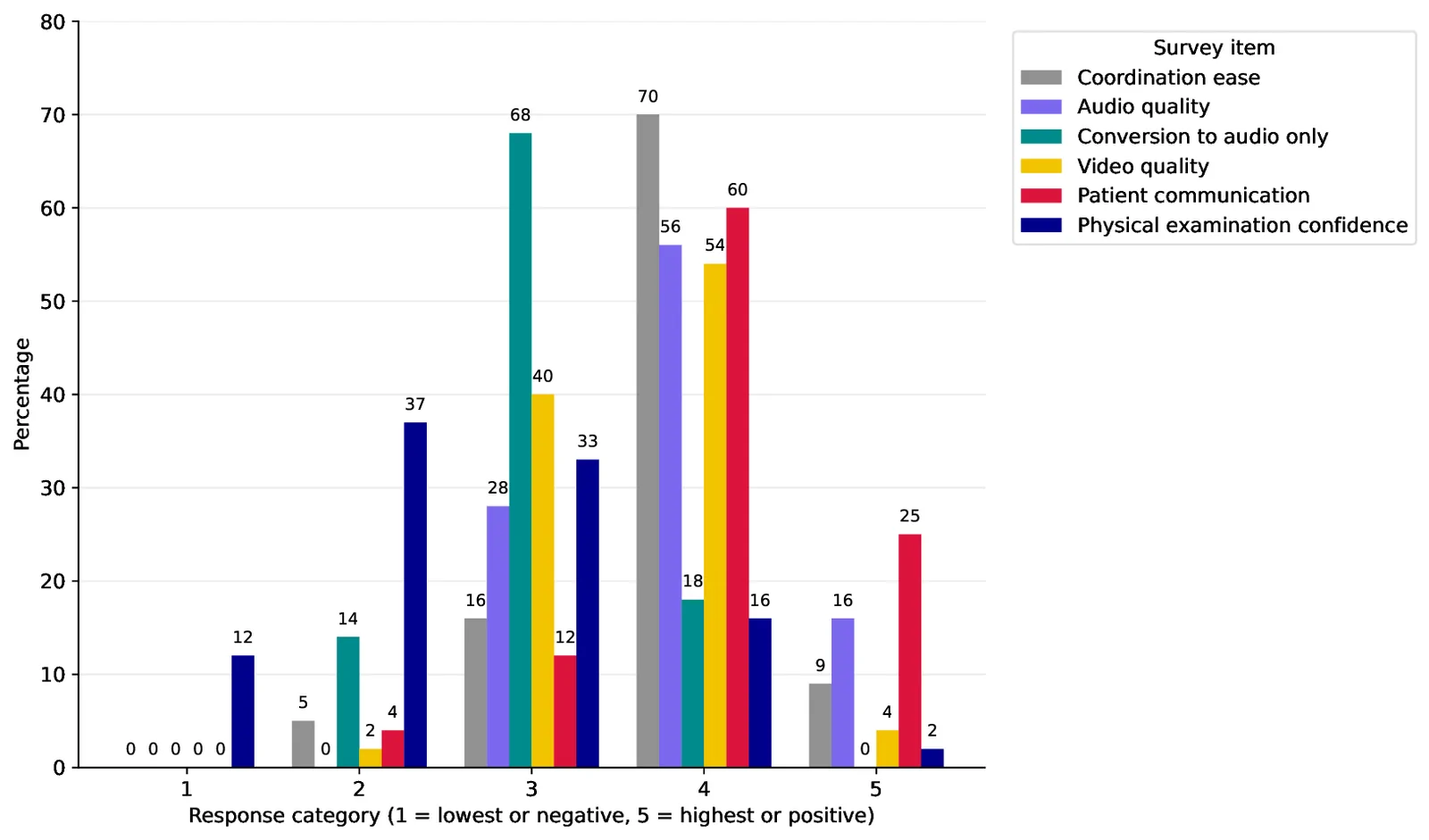
Clinician experience with telehealth across six domains.

Clinician experience with telehealth is summarized in Figure 1 and Table 2. In Figure 1, responses were reported on 5-point Likert scales. For coordination, audio quality, video quality, communication, and physical examination confidence, higher scores indicated more favorable responses. For audio-only conversion, higher scores indicated more frequent conversion. Most respondents reported that coordinating a telehealth visit was easy or very easy (45/57, 78.9%; 95% CI 66.7%‐87.5%). Audio quality was rated good or very good by 41 of 57 (71.9%; 95% CI 59.2%‐81.9%) respondents, while video quality was rated good or very good by 33 of 57 (57.9%; 95% CI 45.0%‐69.8%) respondents. Technical issues were common: 49 of 57 (86%; 95% CI 74.7%‐92.7%) clinicians reported at least occasional conversion to audio-only visits due to audiovisual problems, including 10 (17.5%; 95% CI 9.8%‐29.4%) clinicians who reported frequent conversion.

**Table 2.** Detailed survey responses for telehealth experience and visit modality decision factors^a^.

Domain or survey item	Response summary	Responses, n (%)	95% CI
Telehealth experience (n=57)			
Telehealth coordination	Easy or very easy	45 (78.9)	66.7‐87.5
Audio quality	Good or very good	41 (71.9)	59.2‐81.9
Video quality	Good or very good	33 (57.9)	45.0‐69.8
Audio-only conversion due to technical issues	Occasionally, frequently, or very frequently	49 (86)	74.7‐92.7
Patient communication by telehealth	Somewhat or extremely satisfied	48 (84.2)	72.6‐91.5
Confidence in telehealth physical examination	Moderate or high confidence	29 (50.9)	38.3‐63.4
Visit modality decision factors (n=57)			
Reviewed patient address before visit	Yes	22 (38.6)	27.1‐51.6
Patient distance influenced decision to offer telehealth	Yes	45 (78.9)	66.7‐87.5
Need for physical examination influenced decision to offer telehealth	Yes	53 (93)	83.3‐97.2
Race or ethnicity influenced decision to offer telehealth	Yes	0 (0)	0.0‐6.3
Primary language influenced decision to offer telehealth	Yes	25 (43.9)	31.8‐56.7
Interpreter availability and coordination for telehealth	Somewhat or extremely satisfied	12 (21.1)	12.5‐33.3
Interpreter availability and coordination for telehealth	Somewhat or extremely dissatisfied	31 (54.4)	41.6‐66.6
Likelihood of offering telehealth by patient age (y), (n=57)			
Patients younger than 65 years	Somewhat or extremely likely	34 (59.6)	46.7‐71.4
Patients aged 65-75 years	Somewhat or extremely likely	31 (54.4)	41.6‐66.6
Patients aged 75-85 years	Somewhat or extremely likely	28 (49.1)	36.6‐61.7
Patients older than 85 years	Somewhat or extremely likely	24 (42.1)	30.2‐55.0
Specialty-specific confidence in telehealth^b^			
Surgical oncology: preoperative (n=21)	Moderate or high confidence	11 (52.4)	32.4‐71.7
Surgical oncology: postoperative (n=21)	Moderate or high confidence	15 (71.4)	50.0‐86.2
Medical oncology: prechemotherapy (n=18)	Moderate or high confidence	14 (77.8)	54.8‐91.0
Medical oncology: during chemotherapy (n=17)	Moderate or high confidence	13 (76.5)	52.7‐90.4
Radiation oncology: preradiation (n=16)	Moderate or high confidence	14 (87.5)	64.0‐96.5
Radiation oncology: during radiation (n=16)	Moderate or high confidence	15 (93.8)	71.7‐98.9

a Percentages may not total 100 due to rounding. 95% CIs were calculated using the Wilson method. Moderate or high confidence includes responses indicating at least moderate confidence.

b For specialty-specific confidence items, “not applicable” responses were excluded from denominators.

Despite these technical limitations, communication satisfaction was high. Of 57 respondents, 48 (84.2%; 95% CI 72.6%‐91.5%) were somewhat or extremely satisfied with patients’ ability to communicate questions and concerns by telehealth. In contrast, confidence in the remote physical examination was more limited: 29 of 57 (50.9%; 95% CI 38.3%‐63.4%) respondents reported moderate or high confidence in performing a physical examination by telehealth, while 28 of 57 (49.1%; 95% CI 36.6%‐61.7%) respondents reported being not confident or not very confident.

### Decision Factors for Offering Telehealth

Factors influencing clinicians’ decisions to offer telehealth are summarized in Table 2. The need for a physical examination was the most frequently reported factor influencing visit modality selection, cited by 53 of 57 (93%; 95% CI 83.3%‐97.2%) clinicians. Patient distance from clinic also strongly influenced modality selection, cited by 45 of 57 (78.9%; 95% CI 66.7%‐87.5%) clinicians. Although distance influenced decision-making for most respondents, only 22 of 57 (38.6%; 95% CI 27.1%‐51.6%) clinicians reported reviewing a patient’s address before a scheduled visit.

**Figure 2.**
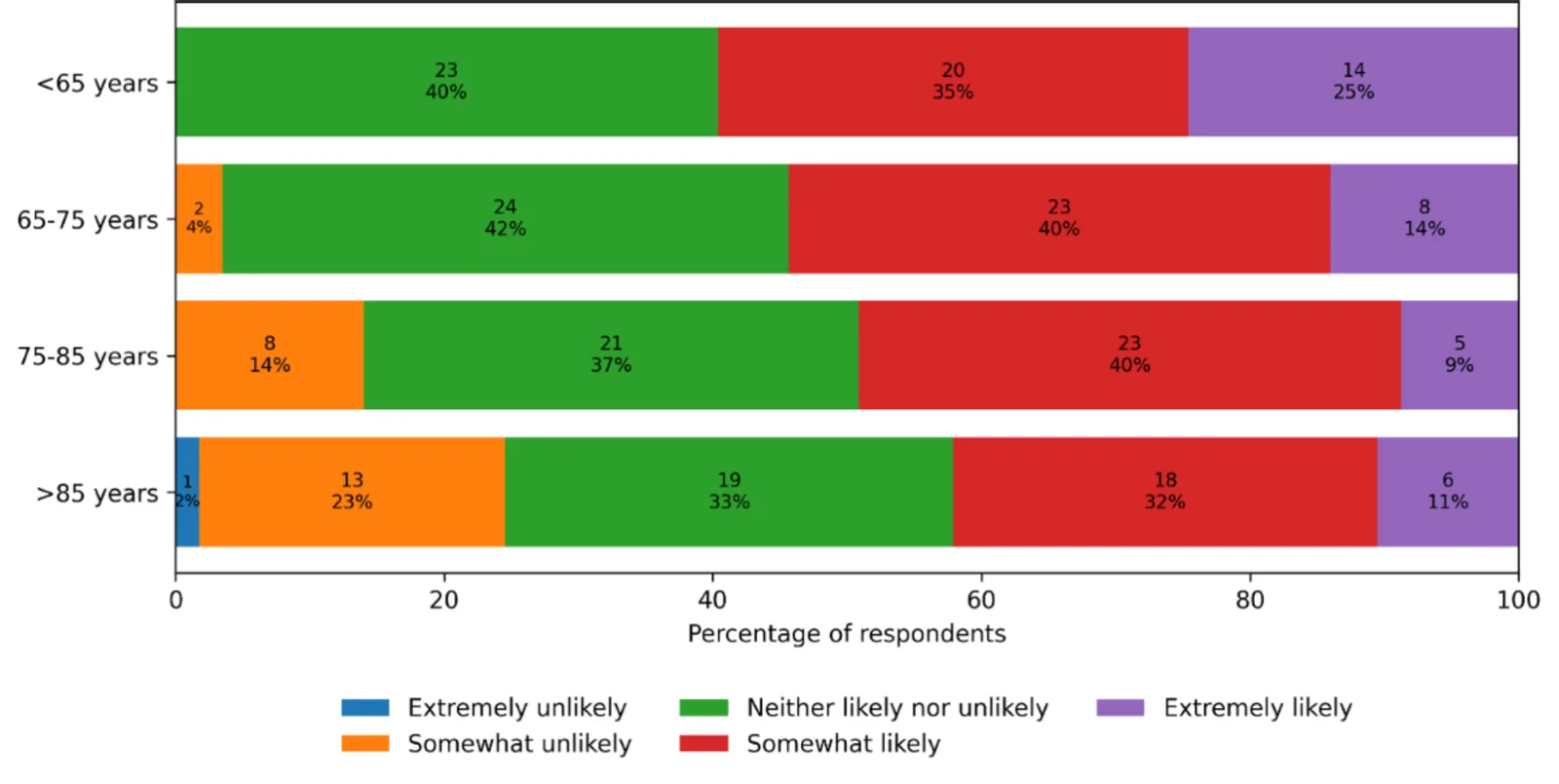
Likelihood of offering telehealth by patient age group. Bars show the distribution of clinician responses across likelihood categories for each patient age group. Numbers within bars indicate respondent counts and percentages. Percentages may not total 100 due to rounding.

Patient age also influenced telehealth decision-making (Figure 2). The proportion of clinicians reporting that they were somewhat or extremely likely to offer telehealth declined across increasing age categories: 34 of 57 (59.6%; 95% CI 46.7%‐71.4%) for patients younger than 65 years, 31 of 57 (54.4%; 95% CI 41.6%‐66.6%) for patients aged 65 to 75 years, 28 of 57 (49.1%; 95% CI 36.6%-61.7%) for patients aged 75 to 85 years, and 24 of 57 (42.1%; 95% CI 30.2%‐55.0%) for patients older than 85 years. This decline across age categories was statistically significant in exploratory ordinal analysis (Friedman test: *P*<.001). No clinician reported that race or ethnicity influenced their decision to offer telehealth. In contrast, 25 of 57 (43.9%; 95% CI 31.8%‐56.7%) clinicians reported that a patient’s primary language influenced their decision to offer telehealth.

Interpreter-related responses were less favorable than other telehealth domains. Only 12 of 57 (21.1%; 95% CI 12.5%‐33.3%) respondents reported being somewhat or extremely satisfied with obtaining an interpreter for telehealth visits, while 31 of 57 (54.4%; 95% CI 41.6%‐66.6%) respondents reported being somewhat or extremely dissatisfied. Open-ended responses emphasized that interpreter integration into telehealth visits was technically and logistically challenging and that in-person interpretation was often preferred for complex conversations.

### Qualitative Themes

Thematic analysis of open-ended responses identified several recurring themes related to telehealth decision-making. When discussing patient distance from the clinic, clinicians emphasized travel burden, cost and time toxicity, access to care, patient preference, and clinical appropriateness. Telehealth was viewed as particularly useful when the visit involved review of laboratory results, imaging, treatment planning, routine follow-up, or postoperative care without a need for physical examination. Representative comments included that telehealth can reduce unnecessary travel and improve follow-up access for patients who live far from the cancer center.

When discussing primary language, clinicians described interpreter use during telehealth visits as cumbersome, time-consuming, and technically unreliable. Several respondents stated that communication was easier when interpreters were present in person, particularly for visits requiring complex counseling or visual explanations. These themes are summarized with representative quotations in Table 3.

**Table 3.** Thematic analysis of qualitative responses regarding patient distance and primary language as factors influencing the decision to offer telehealth.

Theme summary	Representative quotes
Does a patient’s distance from your clinic influence your decision to offer a telehealth visit?	
Cost, time, and financial toxicity	“Telehealth avoids the ridiculous parking fee the university charges our patients.” “Unnecessary time spent in a car or bus can be avoided.”
Clinical judgment and visit type	“I feel more confident in-person for breast patients but will offer telehealth for initial consults if travel is difficult.” “A physical exam is not necessary and they live far away, I offer a telehealth visit.”
Access, compliance, and equity	“I want their distance to not be a barrier for follow-up care.” “It allows better access to care and increases patient compliance.”
Legal and geographic considerations	“I was informed that the patient needs to legally be in California at the time of telehealth visit.”
Patient preference and flexibility	“Patients often try to switch their visit to telemed due to distance or traffic.” “If patient mentions travel time, I consider telehealth.”
Does a patient’s primary language influence your decision to offer a telehealth visit?	
Interpreter use is challenging via telehealth	“Translation services are spotty for telehealth visits at best, and rates of technical difficulties are much higher.” “I need a translator for telehealth so find it very difficult to do that.”
Preference for in-person interpretation	“Interpreters are best in person with actual live humans.” “Understanding each other is much easier if at least some of the people involved are in person.”
Communication barriers and patient comprehension	“I often like to draw figures/pictures for my patients which help them better understand certain concepts.” “More difficult to coordinate translator on video. Communication and connection issues make it hard.”
Impact on visit quality and duration	“It takes longer. Difficult to use an interpreter via telehealth.” “It is cumbersome to request an interpreter over telephone and/or video.”
Workarounds and limitations	“If the patient has a family member who is willing/able to translate, then I am more keen on using a telehealth visit.” “I have not found a good way to do video telehealth with interpreters.”

### Experience by Specialty and Phase of Care

**Figure 3.**
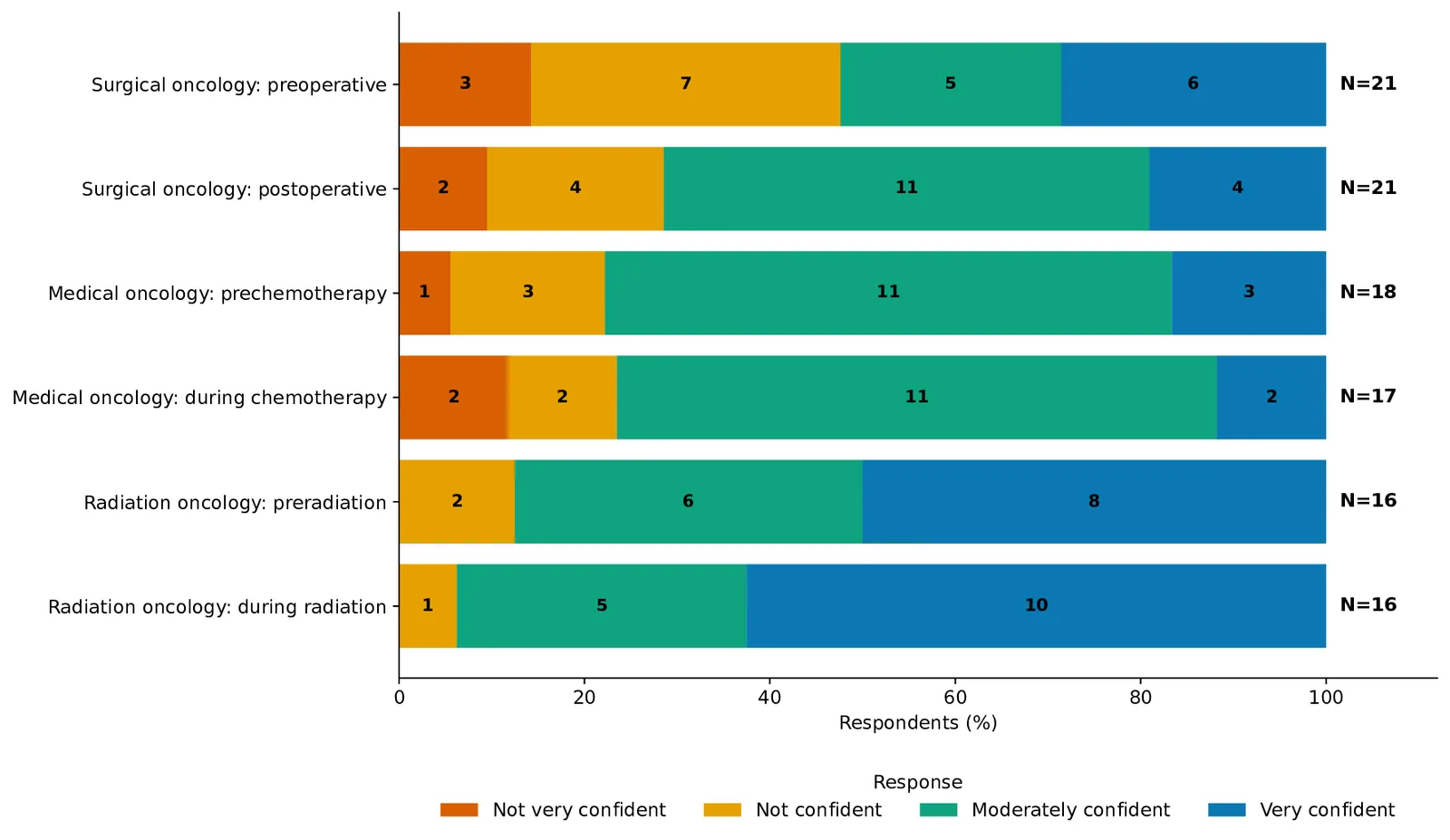
Clinician confidence in telehealth by oncology specialty and phase of care. Bars represent the distribution of provider responses for each specialty-specific clinical scenario. Numbers within bars indicate respondent counts, and denominators on the right indicate the number of applicable respondents for each scenario. “Not applicable” responses were excluded from denominators.

Confidence in telehealth varied across specialty-specific clinical scenarios (Figure 3). Among surgical oncology clinicians, 11 of 21 (52.4%; 95% CI 32.4%‐71.7%) reported moderate or high confidence in assessing patients by telehealth in the preoperative setting, compared with 15 of 21 (71.4%; 95% CI 50.0%‐86.2%) in the postoperative setting. This within-specialty increase was descriptive and was not statistically significant in paired exploratory analysis (McNemar test: *P*=.39).

Among medical oncology clinicians, 77.8% (14/18; 95% CI 54.8%‐91.0%) reported moderate or high confidence in assessing patients by telehealth before chemotherapy. For assessment while receiving chemotherapy, 76.5% (13/17; 95% CI 52.7%‐90.4%) of respondents with nonmissing and applicable responses reported moderate or high confidence. Among radiation oncology clinicians, 87.5% (14/16; 95% CI 64.0%‐96.5%) reported moderate or high confidence before radiation therapy and 93.8% (15/16; 95% CI 71.7%‐98.9%) reported moderate or high confidence during radiation therapy.

In exploratory comparisons across specialty-specific pretreatment scenarios, moderate or high confidence differed across surgical, medical, and radiation oncology clinicians (11/21, 52.4% vs 14/18, 77.8% vs 14/16, 87.5%; chi-square test: *P*=.049). Differences across postoperative, during chemotherapy, and during radiation therapy scenarios were not statistically significant (15/21, 71.4% vs 13/17, 76.5% vs 15/16, 93.8%; chi-square test: *P*=.23).

### Overall Experience

Overall, clinicians viewed telehealth as a useful adjunct to in-person oncology care when used for clinically appropriate visits. Commonly cited advantages included improved access, reduced travel burden, greater flexibility, and support for longitudinal follow-up. Commonly cited areas for improvement included audiovisual reliability, interpreter integration, patient preparation and expectations, and improvements to the clinician-facing telehealth platform. Clinicians consistently emphasized that telehealth should complement rather than replace in-person care, particularly when physical examination or complex communication is required.

## Discussion

### Principal Findings

In this cross-sectional survey of oncology clinicians at an academic cancer center, telehealth was generally viewed as a useful adjunct to traditional in-person care. Clinicians reported high satisfaction with visit coordination and patient communication but identified audiovisual reliability, interpreter integration, and limitations of remote physical examination as important barriers. Confidence in telehealth varied by specialty and phase of care, reflecting differences in clinical workflows and the role of physical examination across surgical, medical, and radiation oncology.

Our findings suggest that clinician confidence with telehealth is context dependent rather than uniform across oncology care. Surgical oncology clinicians reported greater confidence in postoperative than preoperative telehealth assessment, which likely reflects the greater need for physical examination, procedural planning, and initial treatment counseling before surgery. In contrast, medical and radiation oncology clinicians reported relatively high confidence across treatment-related scenarios, where telehealth may be particularly useful for counseling, reviewing laboratory or imaging results, symptom assessment, and longitudinal follow-up. These findings support the concept that telehealth is best viewed as a modality matched to visit type rather than a universal replacement for in-person oncology care.

### Telehealth Decision-Making and Workflow Implications

The need for physical examination and patient distance from the clinic were the most influential factors in clinicians’ decisions to offer telehealth. This reflects a practical balance between clinical appropriateness and patient access. Clinicians described telehealth as a way to reduce travel burden, time toxicity, parking and transportation costs, and barriers to follow-up for patients living far from the cancer center. However, only a minority of respondents reported routinely reviewing a patient’s address before a scheduled visit, suggesting that opportunities exist to make distance-informed telehealth triage more systematic.

Institutions could use these findings to develop clearer workflows for identifying visits that are well suited for telehealth. Examples include standardized visit-type criteria, previsit screening for the need for physical examination, workflows that identify patients with long travel distances, and patient-facing instructions to improve technical readiness before the visit. Embedding these processes into scheduling and electronic health record workflows may help reduce variability in how telehealth is offered while preserving clinician judgment.

### Equity Considerations

Although telehealth has the potential to improve access, our findings also highlight equity concerns. Clinicians were less likely to favor telehealth for older patients and reported substantial difficulty using interpreter services during telehealth visits. These findings are consistent with prior work showing that telehealth adoption may vary by patient characteristics, language, technology access, and health system infrastructure [7,12]. Without intentional workflows, telehealth could inadvertently widen disparities among patients who already face barriers to cancer care.

Several practical interventions may mitigate these risks. Institutions could improve interpreter integration into video visit platforms, develop workflows for previsit interpreter scheduling, provide technical support before visits, and offer patient education materials in multiple languages. For older patients or patients with limited digital access, previsit test calls, caregiver inclusion, simplified login processes, and hybrid models that combine in-person and telehealth visits may help preserve access without compromising care quality. These strategies may be especially important in oncology, where complex counseling and shared decision-making are common.

### Comparison With Prior Literature

Our results align with prior studies demonstrating broad acceptance of telehealth in cancer care while also identifying persistent technical, communication, and patient safety concerns [8,12,13]. Similar to prior work, we found that telehealth use depends on clinical context and specialty-specific workflows [6,13]. Our study adds to this literature by comparing clinician experiences across oncology subspecialties and by evaluating specialty-specific confidence across phases of cancer care. This provides a more nuanced view of where providers perceive telehealth to be most clinically useful and where in-person visits remain important.

### Limitations

This study has several limitations. First, it was conducted at a single academic cancer center, and findings may not generalize to community practices, nonacademic settings, or institutions with different telehealth infrastructure. Second, although we were able to calculate an overall complete response rate of 50% (57/114), response rates varied by specialty, and nonresponse bias remains possible. Third, although the survey was reviewed by investigators for face validity and clinical relevance, it did not undergo formal psychometric validation, cognitive testing, or reliability testing. Fourth, subgroup sizes were modest, particularly for palliative care and for specialty-specific phase-of-care analyses, and exploratory statistical comparisons should therefore be interpreted cautiously.

Fifth, the study focused on clinician experience and did not include patient perspectives, objective visit outcomes, completion rates, or measures of clinical quality. Sixth, tumor-site specialty was not collected. This is important because the role of physical examination and perceived appropriateness of telehealth may differ by disease site, such as breast, genitourinary, gastrointestinal, thoracic, or central nervous system malignancies. Finally, telehealth workflows and reimbursement policies continue to evolve, and clinician experiences may change as technology, interpreter integration, and institutional practices improve.

### Future Directions

Future studies should validate these findings in larger, multi-institutional cohorts and should include both clinician and patient perspectives. Additional work should evaluate disease site–specific telehealth appropriateness, identify visit types that can be safely and effectively conducted virtually, and test interventions to improve interpreter integration, technical reliability, and equitable access. As telehealth reimbursement policies and digital infrastructure continue to evolve, understanding clinician-level experiences across oncology specialties may help guide workflows that maintain care quality while improving patient access.

### Conclusions

Telehealth is a useful adjunct to in-person oncology care across medical, surgical, and radiation oncology, but clinician confidence and perceived utility vary by specialty and phase of care. Our findings support using telehealth as a complementary care modality when physical examination is not essential and when virtual visits can reduce travel burden or improve access. Further integration of telehealth into oncology care will require intentional workflows, improved audiovisual reliability, better interpreter integration, and attention to equitable access for all patients.

## Supplementary material

10.2196/94610 Multimedia Appendix 1 The 27-item Qualtrics survey instrument distributed to oncology providers to assess telehealth experience, visit modality decision factors, interpreter use, likelihood of offering telehealth by patient age, and specialty-specific confidence across phases of cancer care.

## Abbreviations

IRB: institutional review board
