# Supplementary material for: Cancer Clinician Experiences With Telehealth Across Oncology Specialties: Cross-Sectional Survey Study
Source: JMIR Cancer. 2026 Aug 19;12:e94610. doi: 10.2196/94610 (PMC13489505; doi:10.2196/94610)
Supplement: Supplementary file 1 [file cancer-v12-e94610-s001.pdf]

Multimedia Appendix 1. Telehealth Provider Survey Instrument

Cancer Provider Experiences with Telehealth Across Oncology Specialties: Cross-Sectional Survey Study

This appendix reproduces the survey questions and response options. Qualtrics administration URLs, browser timestamps, and export footer artifacts have been removed.

Introduction

IRB: #810808

Welcome to the Telehealth Provider Survey

Thank you for participating in our survey on telehealth in oncology. The COVID-19 pandemic has accelerated the use of telehealth in cancer care, and we aim to understand healthcare providers' experiences and perceptions of this shift.

Survey Purpose: We want to learn how factors such as patient demographics and technology influence your decision to offer telehealth. Your responses will help improve telehealth services in oncology care.

What to Expect: This survey will take 10-15 minutes. All responses will remain confidential.

Thank you for your time and valuable input!

Demographics Block

Please enter your name:

What is your age?

What is your specialty?

- Radiation Oncology
- Medical Oncology
- Surgical Oncology
- Palliative Care

What is your sex?

- Male
- Female
- Non-binary
- Prefer not to say

What is your race?

- White
- Black or African American
- Native American or Alaska Native
- Asian
- Native Hawaiian or Pacific Islander
- Other

Please specify:

What is your ethnicity?

- Hispanic
- Non-Hispanic

What is your professional role?

- Physician
- Advanced Practice Provider (APP)
- Other

Please specify:

Experience With Telehealth Block

How difficult or easy is it coordinate a telehealth visit for your patient?

- Very difficult
- Difficult
- Neither easy nor difficult
- Easy
- Very easy

How is the audio quality of your telehealth visits?

- Very Poor
- Poor
- Average
- Good
- Very Good

How is the video quality of your telehealth visits?

- Very Poor
- Poor
- Average
- Good
- Very Good

How frequently do you have to convert to an audio-only visit based on technical difficulties with the audio/visual?

- Never
- Rarely
- Occasionally
- Frequently
- Very Frequently

How satisfied are you with patients' ability to communicate their questions and concerns by telehealth?

- Extremely dissatisfied
- Somewhat dissatisfied
- Neither satisfied nor dissatisfied
- Somewhat satisfied
- Extremely satisfied

How confident are you in your physical examination by telehealth?

- Not Very Confident
- Not Confident
- Moderately Confident
- Confident
- Very Confident

Decision Factors Block

Do you review a patient's address prior to their scheduled appointment with you?

- No
- Yes

Does a patient's distance from your clinic influence your decision to offer a telehealth visit?

- No
- Yes

If yes, please explain why?

Does the need for a physical examination influence your decision to offer a telehealth visit?

- No
- Yes

Does a patient's race or ethnicity influence your decision to offer a telehealth visit?

- No
- Yes

If yes, please explain why?

Does a patient's primary language influence your decision to offer a telehealth visit?

- No
- Yes

If yes, please explain why?

How satisfied are you with getting an interpreter if necessary for a telehealth visit?

- Extremely dissatisfied
- Somewhat dissatisfied
- Neither satisfied nor dissatisfied
- Somewhat satisfied
- Extremely satisfied

How likely are you to offer a patient a telehealth visit if they are under 65?

- Extremely unlikely
- Somewhat unlikely
- Neither likely nor unlikely
- Somewhat likely
- Extremely likely

How likely are you to offer a patient a telehealth visit if they are between 65-75 years of age?

- Extremely unlikely
- Somewhat unlikely
- Neither likely nor unlikely
- Somewhat likely
- Extremely likely

How likely are you to offer a patient a telehealth visit if they are between 75-85 years of age?

- Extremely unlikely
- Somewhat unlikely
- Neither likely nor unlikely
- Somewhat likely
- Extremely likely

How likely are you to offer a patient a telehealth visit if they are over 85 years of age?

- Extremely unlikely
- Somewhat unlikely
- Neither likely nor unlikely
- Somewhat likely
- Extremely likely

Rank the following from most important to least important when considering whether to offer a patient a telehealth visit. *Rank 1 is the most important and rank 5 is the least important. Choose one rank for each option.*

- Distance from clinic
- Age
- Race / Ethnicity
- Primary Language
- Need for in-person physical examination

For Surgeons Only

How confident are you in assessing a patient by telehealth alone prior to surgery?

- Not very Confident
- Not Confident
- Moderately Confident
- Very Confident
- Not Applicable

How confident are you in assessing a patient by telehealth alone after surgery?

- Not very Confident
- Not Confident
- Moderately Confident
- Very Confident
- Not Applicable

For Medical Oncologists Only

How confident are you in assessing a patient by telehealth alone before chemotherapy?

- Not very Confident
- Not Confident
- Moderately Confident
- Very Confident
- Not Applicable

How confident are you in assessing a patient by telehealth alone when receiving chemotherapy?

- Not very Confident
- Not Confident
- Moderately Confident
- Very Confident
- Not Applicable

For Radiation Oncologists Only

How confident are you in assessing a patient by telehealth alone before radiation therapy?

- Not very Confident
- Not Confident
- Moderately Confident
- Very Confident
- Not Applicable

How confident are you in assessing a patient by telehealth alone during radiation therapy?

- Not very Confident
- Not Confident
- Moderately Confident
- Very Confident
- Not Applicable

Additional Feedback

Do you have any additional feedback about your experience with telehealth within the context of patient care at UC San Diego Health?
